# The effects of a dialogue-based intervention to promote psychosocial well-being after stroke: a randomized controlled trial

**DOI:** 10.1177/0269215520929737

**Published:** 2020-06-10

**Authors:** Line Kildal Bragstad, Ellen Gabrielsen Hjelle, Manuela Zucknick, Unni Sveen, Bente Thommessen, Berit Arnesveen Bronken, Randi Martinsen, Gabriele Kitzmüller, Margrete Mangset, Kari Johanne Kvigne, Katerina Hilari, C Elizabeth Lightbody, Marit Kirkevold

**Affiliations:** 1 Research Center for Habilitation and Rehabilitation Services and Models (CHARM), Institute of Health and Society, University of Oslo, Oslo, Norway; 2 Department of Geriatric Medicine, Oslo University Hospital, Oslo, Norway; 3 Oslo Centre for Biostatistics and Epidemiology, Department of Biostatistics, University of Oslo, Oslo, Norway; 4 Faculty of Health Sciences, Oslo Metropolitan University, Oslo, Norway; 5 Department of Physical Medicine and Rehabilitation, Oslo University Hospital, Oslo, Norway; 6 Department of Neurology, Akershus University Hospital, Lørenskog, Norway; 7 Department of Health and Nursing Sciences, Faculty of Social and Health Sciences, Inland Norway University of Applied Sciences, Elverum, Norway; 8 Department of Health and Care Sciences, Faculty of Health Sciences, UiT, The Arctic University of Norway, Narvik, Norway; 9 Centre for Language and Communication Sciences Research, School of Health Sciences, City, University of London, London, UK; 10 School of Nursing, University of Central Lancashire, Lancashire, UK

**Keywords:** Stroke, rehabilitation, randomized controlled trial, sense of coherence, psychosocial support systems

## Abstract

**Objective:**

To evaluate the effect of a dialogue-based intervention targeting psychosocial well-being at 12 months post-stroke.

**Design:**

Multicenter, prospective, randomized, assessor-blinded, controlled trial with two parallel groups.

**Setting:**

Community.

**Subjects:**

Three-hundred and twenty-two adults (⩾18 years) with stroke within the last four weeks were randomly allocated into intervention group (*n* = 166) or control group (*n* = 156).

**Interventions:**

The intervention group received a dialogue-based intervention to promote psychosocial well-being, comprising eight individual 1–1½ hour sessions delivered during the first six months post-stroke.

**Main measures:**

The primary outcome measure was the General Health Questionnaire-28 (GHQ-28). Secondary outcome measures included the Stroke and Aphasia Quality of Life Scale-39g, the Sense of Coherence scale, and the Yale Brown single-item questionnaire.

**Results:**

The mean (SD) age of the participants was 66.8 (12.1) years in the intervention group and 65.7 (13.3) years in the control group. At 12 months post-stroke, the mean (SE) GHQ-28 score was 20.6 (0.84) in the intervention group and 19.9 (0.85) in the control group. There were no between-group differences in psychosocial well-being at 12 months post-stroke (mean difference: −0.74, 95% confidence interval (CI): −3.08, 1.60). The secondary outcomes showed no statistically significant between-group difference in health-related quality of life, sense of coherence, or depression at 12 months.

**Conclusion:**

The results of this trial did not demonstrate lower levels of emotional distress and anxiety or higher levels of health-related quality of life in the intervention group (dialogue-based intervention) as compared to the control group (usual care) at 12 months post-stroke.

## Introduction

Stroke is one of the leading causes of death and disability in the adult population worldwide.^1^ It may have a devastating effect on people, not only physically, but also emotionally; therefore, it is not surprising that psychosocial well-being may be threatened following stroke. Depressive symptoms, anxiety, general psychological distress, and social isolation are prevalent.^2,3^ About one-third of stroke survivors report depressive symptoms, and 20% report anxiety post-stroke.^4,5^ Psychosocial problems persist over time, and the prevalence of post-stroke depression remains high at 25% in the period from 1 to 5 years post-stroke.^4^ Psychosocial difficulties may significantly impact long-term functioning and quality of life,^6,7^ reduce the effects of rehabilitation services, and lead to higher mortality.^8^

Despite inconclusive evidence,^9,10^ targeted treatments to promote psychosocial adjustment may improve psychosocial well-being.^6,11^ In our work, psychosocial well-being was defined as consisting of a basic mood of contentment, a self-concept characterized by self-acceptance, usefulness, and a belief in one’s abilities. Having social relationships and support, a feeling of loving and being loved in relationships are included in the definition, as well as participation and engagement in meaningful activities beyond oneself.^12,13^ The feasibility work preceding this randomized controlled trial (RCT) suggested that it is possible to promote psychosocial well-being and coping through a dialogue-based intervention.^13–15^

We hypothesized that a dialogue-based intervention would lead to higher levels of psychosocial well-being expressed through lower levels of emotional distress and anxiety at 12 months post-stroke. Secondary hypotheses were that stroke survivors who received the intervention would experience significantly higher levels of health-related quality of life and sense of coherence at 12 months post-stroke.

## Methods

In this study, patient enrollment started in November 2014 and concluded data collection in November 2017. The Regional Committee for Medical and Health Research Ethics, South-East (REC no. 2013/2047) and the Data Protection Officer serving all participating hospitals (Case number: 2014/1026) approved the study. Written informed consent was obtained from all participants. Due to strict regulations and conditions for informed consent enforced by Norwegian law, the data set that supports the findings of this study is not publicly available. A subset of the data may be made available from the corresponding author upon reasonable request. The study was registered with ClinicalTrials.gov (NCT02338869), and the study protocol outlining the full details of the study was published in BMC Psychology in 2018.^15^

The study was a multicenter, prospective, randomized, assessor-blinded, controlled trial with two parallel groups (intervention and standard stroke treatment) and an equal size allocation ratio of 1:1. Eleven hospitals with stroke units or rehabilitation centers in South-Eastern Norway enrolled patients. Eligible participants were adults aged ⩾18 years, had suffered an acute stroke within the last four weeks, were medically stable, had sufficient cognitive functioning to participate, were able to understand and speak Norwegian before stroke onset, and were able to give informed consent. Exclusion criteria were moderate to severe dementia or other serious physical or psychiatric diseases and severe receptive or expressive aphasia.

The sample size was determined based on the primary outcome measure General Health Questionnaire-28 (GHQ-28). The calculations were based on a repeated measures logistic regression model of the binary output variable “normal mood” (GHQ-28 < 5) with two measurements for each patient (i.e. one at six months and one at 12 months).^15^ Based on the results of comparable studies,^11,16^ we deemed an odds ratio of 1.6 or higher between groups (intervention/control) with normal mood after six and 12 months to be clinically relevant. With 80% power across both time points and a significance level α at 0.05, the sample size was estimated to 300 patients (150 per group), which was inflated to a total of 330 to allow for a potential 10% drop-out.^15^

A computer-generated block randomization procedure with blocks of 10 stratified by hospital and with an allocation ratio of 5:5 was used in this study. An assistant independent of the research team prepared opaque randomization envelopes. Two regional trial coordinators carried out the allocation following the baseline assessment. Participants were informed about group allocation immediately. To ensure masking of group allocation at the follow-up assessments, a message was issued from the trial coordinators to participants with a reminder not to reveal their group allocations to the assessors.

The primary and secondary outcomes and measures are presented in Table 1. The primary outcome was psychosocial well-being at 12 months post-stroke. The GHQ-28 measures symptoms of emotional distress.^17,18^ In light of the extensive literature of the high prevalence of emotional distress following stroke, we assumed that well-being in this population would premise the absence of emotional distress. Consequently, in this study, we operationalized psychosocial well-being as lower levels of emotional distress and used the GHQ-28 to measure it. For additional details on the scoring of the GHQ-28 in this study, please refer to the online supplementary material (Supplemental file 1). Clinical characteristics such as stroke classification, side localization of the stroke symptoms, stroke severity, cognitive function, and language difficulties were assessed at the hospital and were collected from the patients’ medical records.

**Table 1.** Outcomes and measures with scoring and time of assessment.

	Measure	Description	Scoring	Assessment^a^
Primary outcome				
Psychosocial well-being	The General Health Questionnaire-28 (GHQ-28)^17,18^	Scaled 28-item self-report questionnaire measuring emotional distress. Four subscales identified in psychometric tests (somatic symptoms, anxiety and insomnia, social dysfunction, and severe depression).^17,19^ Likert scoring, items ranging from 1 to 4. Case scoring, items ranging from 0 to 1.	Range sum Likert scoring: 0–84, lower score indicates lower level of distress. Range sum Case scoring: 0–28, cutoff at 5; <5 indicates normal mood, and ⩾5 indicates low mood.	T1, T2, T3
Secondary outcomes				
Health-related quality of life	Stroke and Aphasia Quality of Life Scale-39 generic stroke version (SAQOL-39g)^20,21^	Self-report 39-item stroke-specific health-related quality-of-life scale. Measures patient’s perspective of stroke’s impact on ‘physical’, ‘psychosocial’, and ‘communication’ domains. Likert scoring, items ranging from 1 to 5.	Range mean score: 1–5 Higher mean score indicates higher functioning; higher quality-of-life score.	T1, T2, T3
Sense of coherence	Sense of Coherence scale (SOC-13)^22^	Self-report questionnaire, 13 items measuring the main concepts in the sense of coherence theory; coherence, meaningfulness and manageability. Likert scoring, ranging from 1 to 5.	Sum range: 13–65. Higher scores indicate a stronger sense of coherence.	T1, T2, T3
Depression	The Yale Brown single-item questionnaire (Yale)^23,24^	Self-reported presence or absence of depression	Yes/No	T1, T2, T3
Characteristics of sample				
Fatigue	Fatigue Questionnaire-2 (FQ-2)^25,26^	Self-reported presence or absence of fatigue. If yes; indication of duration of symptoms.	Yes/No	T1, T2, T3
Aphasia	The Ullevaal Aphasia Screening Test (UAS)^27^	Screening for aphasia. Based on scores and clinical judgment, four categories: (1) no language impairment, (2) mild language impairment, (3) moderate language impairment, and (4) severe language impairment.	Range 0–52, scores <50 indicate pathologic language functioning.^28^	T1
Stroke severity/neurological deficit	National Institutes of Health Stroke Scale (NIHSS)^29^	An 11-item scale used by healthcare providers to objectively quantify the impairment caused by a *stroke*.	Range 0–42. Cutoffs: 0–5 = mild symptoms of stroke, 6–10 = moderate symptoms of stroke, ⩾11 = moderate to severe stroke symptoms.	T0
Cognitive function	Mini Mental State Evaluation (MMSE)^30^	30-point test that is used to measure potential cognitive impairment.	Range: 0–30. Cutoff at 24 to indicate cognitive impairment. A score below 24 indicates cognitive impairment ranging from mild (19–23), moderate (10–18), and severe (⩽9).	T0

a T0: Data from acute phase collected from patient record; T1: baseline assessment at four to six weeks post-stroke; T2: Assessment at six months post-stroke; T3: Assessment at 12 months post-stroke.

Data were collected in-person via structured interviews conducted by trained healthcare professionals (registered nurses and occupational therapists) at baseline, four to six weeks post-stroke (T1), and at six months (T2) and 12 (T3) months post-stroke. The data collectors were blinded to group allocation. The participants’ ages, sexes, living situations, caring responsibilities, previous illnesses and comorbidities, and current rehabilitation services were recorded in addition to the structured outcome measures.

All participants randomized into the study received standard stroke treatment in the acute phase according to the Norwegian stroke treatment guideline.^31^ In Norway, patients with minor stroke are typically discharged home with access to interdisciplinary rehabilitation services in the municipality according to the need and availability of the service. Services typically include physical therapy and/or occupational therapy and/or speech and language therapy and/or home nursing care. Systematic psychosocial follow-up is rarely part of the services provided. Patients with severe stroke are typically discharged to a specialized, in-patient rehabilitation unit for specialized rehabilitation services.

Participants randomized to the intervention group were offered a dialogue-based intervention to promote psychosocial well-being. The intervention consisted of eight individual 1–1½-hour sessions between the participants and a specially trained nurse or occupational therapist (intervention providers). The intervention providers completed a three-day training program to learn how to guide the sessions and how to work with the participants based on the principles outlined in the protocol.^15^ The intervention was delivered in the community, primarily in the participants’ homes. The same intervention provider worked with each participant in all sessions.

In line with the protocol,^15^ the intervention started shortly after randomization; four to eight weeks after stroke onset. It lasted 17 weeks, and the last session was completed within six months post-stroke.^15,32^ A guide of stroke-related topics and work-sheets for each session were supplied as part of the intervention.^15^ The intervention provider and the participant were encouraged to individually adapt the order of topics and the time in-between sessions to suit the needs of the participants. Additional details on theoretical perspectives underpinning the intervention, themes, and content of the intervention are outlined in the protocol.^15^

Implementation fidelity was assessed and previously published as part of the process evaluation of this RCT.^32^ The assessment of implementation fidelity included a separate analysis of intervention adherence. The composite adherence score showed that 117 (80.1%) of the intervention trajectories satisfied the criteria for high-fidelity intervention adherence.^32^

## Statistical analysis

The data were analyzed using an intention-to-treat approach. Missing data were imputed using multiple imputations by chained equations in the Statistical Package for the Social Sciences (SPSS).^33,34^ All reported results of the statistical analyses were pooled across five imputations based on Rubin’s rule.^35^ The statistical software R v3.6.1^36^ with package mitools v2.4 was used to pool the results across all five imputed data sets. For additional details of the imputation model, please see the online supplementary material (Supplemental file 2).

Analyses of the primary and secondary outcomes were performed using logistic regression for binary outcomes and independent and paired samples *t* tests for continuous outcomes. A linear mixed model was used to assess the primary outcome of psychosocial well-being at 12 months post-stroke. Due to the complexity of the final model, we did not use the dichotomized “normal mood” (GHQ-28 < 5) end-point, as it resulted in convergence issues when fitting the binary logistic mixed model. The continuous sum-score based on the Likert-type-scoring of GHQ-28 was used as the dependent variable. The other factors of the model remained the same as in the predetermined statistical analysis plan. The details of the linear mixed model are supplied in the online supplementary files (Supplemental file 3).

Statistical tests were performed with SPSS, version 25.0 for Windows.^37^ All statistical tests were performed as two-sided tests with a significance level of α = 0.05.

## Results

**Figure 1.**
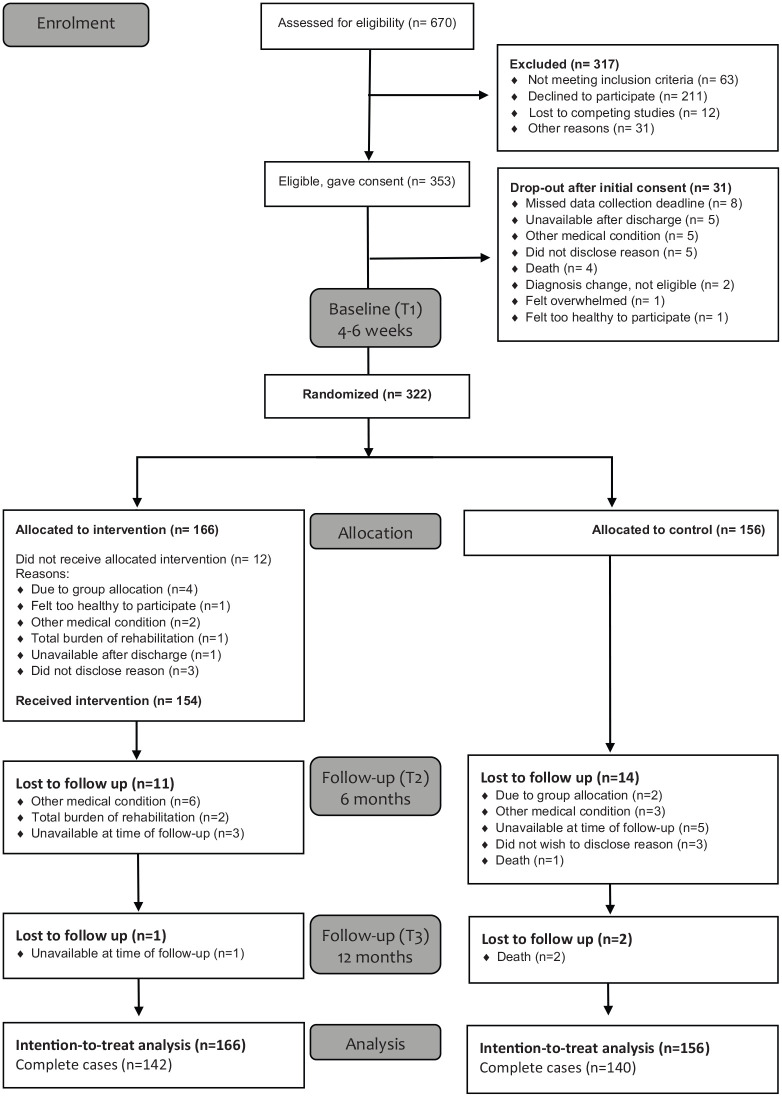
CONSORT participant recruitment and retention flow chart.

The CONSORT flow diagram is presented in Figure 1. Three-hundred and fifty-three (58.2%) of the eligible individuals consented to participate in this study. There were no significant differences in age and sex between individuals who consented and those who did not.^19^ Between consent and the baseline assessment, 31 (8.8%) participants dropped out. Thus, 322 participants were assessed at baseline and subsequently allocated to the intervention group (*n* = 166) or the control group (*n* = 156).

Baseline demographics and clinical characteristics are presented in Table 2, and it shows that the characteristics were similar in both groups except for self-reported depression and stroke classification (infarction or hemorrhage).

**Table 2.** Baseline demographics and clinical characteristics.

	Intervention group (n = 166)	Control group (n = 156)
Baseline demographics		
Age, years, *mean (SD)*	66.8 (12.1)	65.7 (13.3)
Sex		
Female	67 (40.4)	65 (41.7)
Male	99 (59.6)	91 (58.3)
Living conditions		
Living with someone	117 (70.5)	101 (63.7)
Living alone	49 (29.5)	55 (35.3)
Have caring responsibilities	37 (22.3)	36 (23.1)
Underage children	26 (15.7)	22 (14.1)
Spouse or cohabiting partner	10 (6.0)	6 (3.8)
Parents	2 (1.2)	6 (3.8)
Other	2 (1.2)	6 (3.8)
Clinical characteristics		
Stroke classification (*n* = 147; 144)		
Infarction	128 (87.1)	136 (94.4)
Hemorrhage	19 (12.9)	8 (5.6)
Stroke symptom localization (*n* = 142; 136)		
Right	65 (45.8)	56 (41.2)
Left	70 (49.3)	74 (54.4)
Bilateral	7 (4.9)	6 (4.4)
Communication difficulties (*n* = 121; 129)	44 (34.1)	42 (34.7)
Feeling sad or depressed (Yale)	29 (17.5)	43 (27.6)
Feeling fatigued (FQ-2; *n* = 165; 156)	88 (53.3)	87 (55.8)
NIHSS, *median (IQR; n* = 126; 114)	4 (1–7)	2.5 (1–6)
NIHSS categorized scores (*n* = 126; 114)		
Mild (0–5)	85 (67.5)	85 (74.6)
Moderate (6–10)	28 (22.2)	17 (14.9)
Moderate to severe (11+)	13 (10.3)	12 (10.5)
MMSE, *median (IQR)* (*n* = 63; 65)	27 (25–29)	28 (26–30)
UAS (*n* = 163; 156) *median (IQR)*	52 (50–52)	52 (50–52)
Receive one or more rehabilitation services at baseline	114 (68.7)	99 (63.5)
Physical therapy	98 (59.0)	88 (56.4)
Occupational therapy	73 (44.0)	62 (39.7)
Speech and language therapy	30 (18.1)	27 (17.3)
Home nursing care	56 (33.7)	46 (29.5)
Psychologist/psychiatrist	14 (8.4)	11 (7.1)
Other	22 (13.3)	14 (9.0)
Comorbidities		
No reported comorbidities	31 (18.7)	32 (20.5)
Hypertension	71 (42.8)	64 (41.0)
Heart disease	49 (29.5)	39 (25.0)
Diabetes	22 (13.3)	21 (13.5)
Stroke	22 (13.3)	25 (16.0)
Cancer	21 (12.7)	21 (13.5)
Musculoskeletal diseases	21 (12.7)	22 (14.1)
Rheumatic diseases	16 (9.6)	15 (9.6)
Depression	13 (7.8)	16 (10.3)
Gastrointestinal diseases	12 (7.2)	11 (7.1)
Lung disease	8 (4.8)	10 (6.4)
Other reported comorbidities	16 (9.6)	15 (9.6)

SD, standard deviation; IQR, interquartile range; NIHSS, National Institutes of Health Stroke Scale; MMSE, the Mini Mental State Exam; UAS, Ulleval Aphasia Screening.

Comorbidities and rehabilitation services were self-reported by participants. Communication difficulties were self-reported and/or assessed in the acute phase and recorded in patient records. Values are *n* (%) unless stated otherwise.

Results from the between-group comparisons at 12 months post-stroke for primary and secondary outcomes are presented in Table 3. There were no between-group differences in psychosocial well-being at 12 months post-stroke (mean difference: −0.74, 95% confidence interval (CI): −3.08, 1.60).

**Table 3.** Primary and secondary outcomes with between-group differences at 12-month follow-up, by group.

Outcomes	Intervention group (*n* = 166)			Control group (*n* = 156)			Between-group differences at 12 months (T3)	
	T1	T2	T3	T1	T2	T3		*P-*value^a^
GHQ-28 (<5)^b^ (*N* (%))	50 (30.1)	99 (59.6)	109 (65.7)	46 (29.5)	93 (59.6)	103 (66.0)	0.98 (0.62, 1.57)	0.946^c^
GHQ-28 (sum, range: 0–84^b^; mean (SE))	25.9 (0.84)	21.2 (0.83)	20.6 (0.84)	28.5 (0.98)	21.5 (0.89)	19.9 (0.85)	−0.74 (−3.08, 1.60)	0.537^d^
Somatic symptoms (range: 0–9; mean (SE))^e^	2.4 (0.18)	1.8 (0.17)	1.8 (0.15)	2.8 (0.20)	1.8 (0.17)	1.9 (0.15)	0.10 (−0.31, 0.52)	0.618^d^
Anxiety and insomnia (range: 0–33; mean (SE))^e^	8.3 (0.43)	7.1 (0.40)	7.3 (0.43)	9.0 (0.49)	7.4 (0.46)	7.0 (0.45)	−0.30 (−1.55, 0.95)	0.634^d^
Social dysfunction (range: 0–30; mean (SE))^e^	14.8 (0.38)	11.5 (0.40)	10.8 (0.36)	15.7 (0.42)	11.4 (0.38)	10.4 (0.32)	−0.36 (−1.32, 0.60)	0.457^d^
Severe depression (range: 0–12; mean (SE))^e^	0.5 (0.10)	0.7 (0.13)	0.8 (0.13)	0.9 (0.17)	0.9 (0.15)	0.6 (0.11)	−0.18 (−0.52, 0.17)	0.314^d^
Feeling sad or depressed (Yale; *N* (%))^b^	29 (17.4)	37 (22.3)	38 (22.9)	43 (27.6)	36 (23.1)	37 (23.7)	0.96 (0.55, 1.68)	0.890^[Table-fn table-fn9-0269215520929737]c^
Sense of coherence (SOC-13, sum, range: 13–65; mean (SE))^b^	50.6 (0.42)	50.2 (0.58)	50.6 (0.62)	50.4 (0.47)	50.5 (0.52)	51.0 (0.56)	0.43 (−1.09, 1.94)	0.581^d^
Quality of life (SAQOL-39g, mean, range: 1–5; mean (SE))^b^	4.30 (0.04)	4.38 (0.05)	4.36 (0.04)	4.24 (0.05)	4.37 (0.04)	4.43 (0.04)	0.06 (−0.04, 0.17)	0.247^d^
Physical domain (range: 1–5; mean (SE))^b^	4.24 (0.07)	4.52 (0.06)	4.50 (0.05)	4.21 (0.08)	4.52 (0.05)	4.57 (0.05)	0.06 (−0.07, 0.20)	0.364^d^
Communication domain (range: 1–5; mean (SE))^b^	4.75 (0.04)	4.76 (0.03)	4.73 (0.04)	4.74 (0.05)	4.79 (0.03)	4.79 (0.04)	0.06 (−0.05, 0.16)	0.271^d^
Psychosocial domain (range: 1–5; mean (SE))^b^	3.90 (0.06)	3.86 (0.07)	3.85 (0.07)	3.76 (0.06)	3.79 (0.08)	3.93 (0.07)	0.07 (−0.11, 0.25)	0.447^d^

GHQ, General Health Questionnaire; SE, standard error; SOC, Sense of Coherence scale.

T1: Baseline assessment at four to six weeks post-stroke, T2: Assessment at six months post-stroke immediately after intervention, T3: Assessment at 12 months post-stroke.

a Between-group differences at T3.

b Reporting pooled results of imputed data.

c Logistic regression (odds ratio (OR) (95% confidence interval (CI)).

d Independent samples *t* test (mean difference (95% CI)).

e Reporting pooled results of imputed data, new factor structure sum score.

The secondary outcomes showed no statistically significant between-group difference in depression, sense of coherence, or health-related quality of life at 12 months (Table 3). Self-reported depression showed no between-group difference at 12 months (OR: 0.96, 95% CI: 0.55, 1.68). Sense of coherence scores appeared to be stable in both groups throughout the study trajectory. The overall health-related quality of life improved across the trajectory, but there was no statistically significant difference between the intervention and control groups at 12 months (mean difference 0.06, 95% CI: −0.04, 0.17; Table 3).

The results of the linear mixed model analysis are displayed in Table 4. This analysis showed that the fixed effect of time was negative for both six months and 12 months, which implies a reduced GHQ-28 score overall compared to the baseline, indicating a higher level of psychosocial well-being at six months and 12 months post-stroke relative to the baseline (Table 4). In addition, five other explanatory variables had statistically significant fixed effects influencing the GHQ-28 scores.

**Table 4.** Linear mixed model showing fixed effect coefficients.

	Coefficient	SE	95% CI		*P-*value
			Lower	Upper	
*Intercept*	*54.551*	*3.472*	*47.746*	*61.355*	*<0.001*
Time					
Baseline (Ref.)					
Six months post-stroke	−5.648	0.560	−6.745	−4.551	<0.001
12 months post-stroke	−6.490	0.588	−7.642	−5.338	<0.001
Group allocation					
Control group (Ref.)					
Intervention group	−0.956	0.622	−2.175	0.264	0.125
Sex					
Female (Ref.)					
Male	0.124	0.647	−1.145	1.393	0.848
Age at admission	−0.027	0.024	−0.073	0.019	0.249
Stroke classification					
Infarction (Ref.)					
Hemorrhage	0.804	1.131	−1.414	3.021	0.477
Stroke symptom localization					
Right (Ref.)					
Left	0.390	0.673	−0.940	1.719	0.563
Bilateral	1.433	2.022	−2.539	5.405	0.479
Stroke severity (NIHSS)	0.117	0.097	−0.075	0.309	0.228
Live with partner or other	−0.183	0.671	−1.499	1.133	0.785
Comorbidity	1.792	0.716	0.388	3.196	0.012
Rehabilitation services	0.798	0.617	−0.410	2.007	0.195
Caring responsibilities	2.599	0.873	0.889	4.309	0.003
Depression (Yale)	5.514	0.951	3.650	7.377	<0.001
Fatigue (FQ-2)	4.091	0.644	2.829	5.352	<0.001
Sense of coherence (SOC-13)	−0.638	0.058	−0.753	−0.524	<0.001

SE, standard error; CI, confidence interval; NIHSS, National Institutes of Health Stroke Scale; FQ, Fatigue Questionnaire; SOC, Sense of Coherence scale.

Dependent variable: GHQ-28, sum-score range from 0 to 84 (Likert-type scoring). *N* = 322.

Higher scores on sense of coherence were associated with lower GHQ-28 scores, indicating that higher sense of coherence scores were associated with higher levels of psychosocial well-being. Reporting additional comorbidities, caring responsibilities, fatigue, and depression was associated with higher GHQ-28 scores, which indicated lower psychosocial well-being. Adjusted for all factors in the linear mixed model, the intervention group scored lower (mean difference: −0.96 points, 95% CI: −2.18, 0.26) on GHQ-28 compared to the control group; however, the between-group differences were not statistically significant (Table 4).

## Discussion

Contrary to our hypotheses, the results of this trial did not demonstrate at the specified statistical significance level that the participants in the intervention group experienced higher levels of psychosocial well-being and lower levels of depressive symptoms and anxiety than participants in the control group at 12 months post-stroke. Nor did the secondary outcomes show statistically significantly higher levels of sense of coherence or higher levels of health-related quality of life in the intervention group compared with the control group at 12 months post-stroke.

In the following, we will highlight possible reasons for the statistically non-significant results in this RCT, drawing on the results of a comprehensive process evaluation of the RCT and existing research to interpret the outcomes of the trial.^32,38^ Plausible explanations may include flaws in the underlying theoretical assumptions or characteristics of the intervention, the timing of the intervention, the standard care provided to the intervention and control groups, the sample of participants enrolled, or the outcome measures.

Based on Antonovsky’s theory of sense of coherence,^22^ we assumed that an important active ingredient in the intervention would be to support the participants’ perceptions of their lives as comprehensible, manageable, and meaningful. We anticipated that the intervention would foster understanding and re-creation of meaning through narrative dialogue and that the intervention provider could support the participants’ coping efforts and development of new life skills through the guided self-determination problem-solving approach.^13,14^

Antonovsky framed sense of coherence as a stable trait that may to some degree be dynamic with fluctuations in periods of threatening life events.^22^ Others have shown that sense of coherence is less stable over time than Antonovsky assumed.^39^ We hypothesized that the intervention would be able to influence the participants’ sense of coherence after a life-threatening event such as stroke and that a higher sense of coherence would lead to higher levels of psychosocial well-being.

This twofold hypothesis was only supported in part. The lack of differences within groups over time and between the intervention and control group does not support the notion that the intervention succeeded in influencing the levels of sense of coherence. The results of the study suggest that this intervention did not influence sense of coherence and that it is a stable construct. However, the results support the notion that a higher sense of coherence is important in the promotion of psychosocial well-being.

This knowledge may be important to clinicians who need to be able to identify stroke patients who need extra attention with regard to promoting psychosocial well-being. It may be advisable to screen for sense of coherence during the early post-stroke phase to identify those with lower sense of coherence, who may be more vulnerable to lower psychosocial well-being.

Another assumption made in this intervention was that it would be possible to prevent depression that manifested after stroke due to the increased stress and chaos of trying to cope with the post-stroke changes.^3^ For some participants, the intervention may have led to decreasing stress and for some to potentially increasing it, depending on their existing stress levels. If the participants did not experience increased stress or challenges in coping in this phase of their adjustment process, we need to consider if the focus on psychosocial challenges in the intervention may have increased rather than decreased their stress. In the future, screening for distress at baseline may be advisable in order to explore whether the intervention may be more appropriate for those with some level of existing stress/distress.

Based on assumptions that early rehabilitation efforts are important to promote psychosocial well-being,^3,13^ the intervention in this trial was designed to be delivered over a period of five months starting four to six weeks post-stroke and concluding within six months post-stroke.^13,15^ The intervention period coincides with a period in which spontaneous functional recovery may peak^40,41^ and overlaps with a period of comprehensive physical rehabilitation within Norwegian stroke services.^31^ The psychosocial intervention provided to the intervention group may not have made a discernable impact in this context with substantial rehabilitation efforts within the regular healthcare services.

At baseline, participants in both groups reported high scores on the Stroke and Aphasia Quality of Life Scale-39g (Table 3). Although these scores may seem to imply ceiling effects suggesting limited room for improvement, the minimally important difference on the Stroke and Aphasia Quality of Life Scale-39g has been reported as 0.21.^42^ Therefore, despite high baseline scores, there was still room for improvement in health-related quality of life in this group of participants.

The participants received substantial rehabilitation services as part of their standard stroke treatment. At baseline, 114 (68.7%) participants in the intervention group and 99 (63.5%) participants in the control group received one or more rehabilitation services, most frequently physical therapy. At 12 months, the proportion was still high: 70 (42.3%) participants in the intervention group and 66 (42.1%) participants in the control group.

Earlier theoretical work has shown that the physical recovery, daily life adaptation, and normalization, as well as biographical adjustment, occurs simultaneously throughout the first 12 months of the adjustment process after a stroke.^41^ However, the focus on physical recovery is more pronounced in the beginning, while the focus on psychosocial issues such as biographical adjustment gains emphasis later in the trajectory. Introducing this intervention on top of the natural recovery and rehabilitation processes may not have added to the adjustment process, or the participants may have been more focused on other parts of their adjustment than that of a psychosocial nature.

It is important to consider whether the extra attention given to the psychosocial issues in the intervention group came at an inappropriate time in the participants’ stroke recovery and whether we may have increased the awareness on psychosocial difficulties rather than prevented them. Other studies have shown successful results in promoting normal moods when introducing early psychosocial support by providing motivational interviewing to support and build patients’ motivation to adjust and adapt to having had a stroke.^11^

In the study of Watkins and colleagues,^11,16^ motivational interviewing aimed to promote self-efficacy. The patients raised the issues they wanted to discuss themselves instead of having topics outlined for each meeting.^16^ Compared to the theoretical assumptions of anticipated active ingredients of the intervention tested in this RCT, motivational interviewing may have been more aligned with the patients’ phase of adjustment and more aligned with their focus on, that is, getting well or frustration in this early adjustment phase.^43^ The focus on patient-initiated discussion themes rather than the pre-specified themes related to psychosocial issues may have supported their adjustment to a greater degree than in the intervention tested in this RCT.

The feasibility work done during the development of the intervention showed that participants found the intervention helpful; however, it failed to clearly identify specific patient groups who would potentially benefit from this intervention.^13,14,44,45^ Wide inclusion criteria were applied in the RCT, which may have inadvertently resulted in the enrolment of participants who did not particularly need this kind of intervention. The process evaluation that was conducted alongside the trial showed that not all participants expected a personal benefit and that a key motivation to participate was to contribute to research and to help other stroke survivors.^38^ Despite this observation, the majority of the participants who participated in the qualitative interviews as part of the process evaluation found the intervention useful and found that it facilitated their post-stroke adjustments.^38^

Some participants in the control group reported that the assessment interviews facilitated reflection and adjustment, and some indicated that allocation to the control group and the themes raised in the assessment interviews influenced their help-seeking behavior outside the trial.^19^

It is still important to identify subgroups of the stroke population who might benefit from a psychosocial intervention to promote psychosocial well-being. Patients who reported depressive symptoms, fatigue, comorbidities, and caring responsibilities were prone to lower levels of psychosocial well-being in this study. Earlier studies have shown that emotional distress at one month post-stroke, higher stroke severity, and communication impairments predict emotional distress during the first six months post-stroke.^46^

Studies exploring predictors of emotional distress and well-being in a longer post-stroke perspective have found that higher age (>65 years), independence in mobility, having social support, and being employed are important predictors of well-being.^47^ Conversely dependency in activities of daily living (i.e. toileting) predict emotional distress two to five years post-stroke.^47^ Identifying patients with the characteristics identified in this and other studies may be especially important in clinical settings to identify those who may need closer attention and follow-up with regard to psychosocial well-being.

There is a need to consider whether the chosen outcome measures were appropriate to detect the kind of change the intervention targeted. The change in emotional distress in both the intervention and control groups across the trajectory indicated that the GHQ-28 was sensitive to change. There was a substantial increase in the proportion of participants with GHQ-28 scores <5 in both groups. Furthermore, the level of improvement exceeded the findings in a similar study in which motivational interviewing was provided post-stroke.^11,16^ However, the sensitivity of the GHQ-28 does not necessarily mean it was the most suitable outcome measure in this study.

The intervention was aimed at promoting psychosocial well-being. Thus, using an instrument that measured breaks in normal function and presence of emotional distress and reduction in depressive symptoms to enable comparison with similar studies may not have been an ideal choice. Including a measure that targeted the positive concept of well-being more directly, such as the Warwick–Edinburgh Mental Well-being Scales, could have strengthened the study. This scale was developed to enable the measuring of mental well-being in the general population and to enable the evaluation of interventions that aim to improve mental well-being.^48^

Based on the definition of psychosocial well-being used in the development of this intervention, including outcome measures that assess participation in meaningful activities may have added important data to evaluate the outcomes of the intervention. The lack of such an outcome measure was a limitation to this study. Additional outcome measures for participation and well-being should be explored in future research.

A strength in this study was the systematic development and feasibility testing of the intervention prior to full-scale effectiveness tests in this RCT.^13,14^ The trial was conducted in a rigorous manner following the Consolidated Standards of Reporting Trials (CONSORT) statement.^49^

In addition, the comprehensive process evaluation, including the evaluation of implementation fidelity that was conducted alongside the trial,^32^ was an important advantage in documenting the trial implementation and in understanding trial outcomes.

All intervention providers and assessors participating in the study were required to complete training prior to their participation, which was important in establishing uniform delivery of the intervention and the assessment interviews. Completing intervention sessions with parallel goals of individualization and uniform delivery may, however, have been a limitation in this study. Participating in supervision sessions was voluntary for intervention providers, and the follow-ups of the assessors were also based on a voluntary and as-needed basis. In retrospect, mandatory follow-up and supervision may have been warranted to assure uniform delivery of the intervention and uniform assessment across the study trajectory.

Another limitation in this study was the difficulties in enrolling patients with more severe stroke symptoms and aphasia who were presumably more vulnerable to psychosocial problems. However, the sample included in this study represents the largest group of stroke patients admitted to hospitals in Norway.^19^ The nurses and occupational therapists who enrolled participants reported that it was difficult to assess whether patients with aphasia were able to consent. Ensuring an informed consent was perceived to be too time-consuming in the clinical setting, resulting in few participants with aphasia.

Furthermore, enrolment personnel found it difficult to approach the patients with more severe stroke during the short time that they were treated in the stroke unit. These challenges emphasized the need for dedicated personnel who were not involved in other clinical duties while simultaneously enrolling patients to the trial. For future studies, it may be advisable to enroll patients directly from the community and from rehabilitation units providing subacute care to reach a broader group of patients with more severe impairments.

This study showed that certain subgroups (patients reporting depressive symptoms, fatigue, comorbidities, and caring responsibilities) were prone to lower levels of psychosocial well-being. The results also support the notion that a higher sense of coherence is important in the promotion of psychosocial well-being. This may inform inclusion criteria and screening for certain vulnerabilities when enrolling participants in future research.

The results in this study suggest that more research is needed to explore the relationships between psychosocial well-being, sense of coherence, and the process of meaning-making and adjustment following an acute stroke. Additional mechanisms, such as the impact of resilience should be taken into account. Furthermore, exploring these relationships must include the use of more adequate instruments to measure psychosocial well-being.

With respect to clinical practice, there is insufficient evidence to support the implementation of the intervention in its current form based on the outcome measures used in this RCT. However, the inclusion criteria in this study may have been too wide, and further research is needed to confirm whether certain subgroups of stroke patients may benefit from such a psychosocial intervention and at what time post-stroke such an intervention may be appropriate.

**Clinical Messages**

- The dialogue-based intervention implemented in this RCT did not lead to lower levels of emotional distress and anxiety at 12 months post-stroke.
- The intervention did not lead to higher levels of health-related quality of life or higher sense of coherence at 12 months post-stroke.
- Based on the outcome measures used in this study, there is insufficient evidence to support implementation of the intervention in its current form.

## Supplemental Material

SupplementalFile-1-ScoringGHQ-28 – Supplemental material for The effects of a dialogue-based intervention to promote psychosocial well-being after stroke: a randomized controlled trial Click here for additional data file. Supplemental material, SupplementalFile-1-ScoringGHQ-28 for The effects of a dialogue-based intervention to promote psychosocial well-being after stroke: a randomized controlled trial by Line Kildal Bragstad, Ellen Gabrielsen Hjelle, Manuela Zucknick, Unni Sveen, Bente Thommessen, Berit Arnesveen Bronken, Randi Martinsen, Gabriele Kitzmüller, Margrete Mangset, Kari Johanne Kvigne, Katerina Hilari, C Elizabeth Lightbody and Marit Kirkevold in Clinical Rehabilitation

SupplementalFile-2-Imputation – Supplemental material for The effects of a dialogue-based intervention to promote psychosocial well-being after stroke: a randomized controlled trial Click here for additional data file. Supplemental material, SupplementalFile-2-Imputation for The effects of a dialogue-based intervention to promote psychosocial well-being after stroke: a randomized controlled trial by Line Kildal Bragstad, Ellen Gabrielsen Hjelle, Manuela Zucknick, Unni Sveen, Bente Thommessen, Berit Arnesveen Bronken, Randi Martinsen, Gabriele Kitzmüller, Margrete Mangset, Kari Johanne Kvigne, Katerina Hilari, C Elizabeth Lightbody and Marit Kirkevold in Clinical Rehabilitation

SupplementalFile-3-LMM – Supplemental material for The effects of a dialogue-based intervention to promote psychosocial well-being after stroke: a randomized controlled trial Click here for additional data file. Supplemental material, SupplementalFile-3-LMM for The effects of a dialogue-based intervention to promote psychosocial well-being after stroke: a randomized controlled trial by Line Kildal Bragstad, Ellen Gabrielsen Hjelle, Manuela Zucknick, Unni Sveen, Bente Thommessen, Berit Arnesveen Bronken, Randi Martinsen, Gabriele Kitzmüller, Margrete Mangset, Kari Johanne Kvigne, Katerina Hilari, C Elizabeth Lightbody and Marit Kirkevold in Clinical Rehabilitation

Suppl_Table_S1 – Supplemental material for The effects of a dialogue-based intervention to promote psychosocial well-being after stroke: a randomized controlled trial Click here for additional data file. Supplemental material, Suppl_Table_S1 for The effects of a dialogue-based intervention to promote psychosocial well-being after stroke: a randomized controlled trial by Line Kildal Bragstad, Ellen Gabrielsen Hjelle, Manuela Zucknick, Unni Sveen, Bente Thommessen, Berit Arnesveen Bronken, Randi Martinsen, Gabriele Kitzmüller, Margrete Mangset, Kari Johanne Kvigne, Katerina Hilari, C Elizabeth Lightbody and Marit Kirkevold in Clinical Rehabilitation
