# Supplementary material for: The effects of a dialogue-based intervention to promote psychosocial well-being after stroke: a randomized controlled trial
Source: Clin Rehabil. 2020 Jun 10;34(8):1056–71. doi: 10.1177/0269215520929737 (PMC7372590; doi:10.1177/0269215520929737)
Supplement: SupplementalFile-1-ScoringGHQ-28 – Supplemental material for The effects of a dialogue-based intervention to promote psychosocial well-being after stroke: a randomized controlled trial [file SupplementalFile-1-ScoringGHQ-28.pdf]

Scoring of primary outcome measure, GHQ-28.

The primary outcome in this study was emotional distress at 12 months post-stroke measured by the General Health Questionnaire-28 (GHQ-28) [1, 2]. The GHQ-28 is a scaled 28-item self-report questionnaire designed to identify minor psychiatric disorders in the general population within a primary care setting [3]. The items of the questionnaire are phrased to assess the respondent's current state (past two weeks) and focus on breaks in normal function. The Likert scoring method of 0, 1, 2, and 3 [3] with a calculated total sum score (minimum 0, maximum 84) was used in the primary analysis. A lower GHQ-28 score indicates a lower level of distress, interpreted as a higher level of psychosocial well-being. To enable comparison with similar studies, a sum score (minimum 0, maximum 28) using the GHQ-scoring of 0, 0, 1, and 1 [3] was constructed and dichotomized with a cutoff at 5; < 5 indicates normal mood, and ≥ 5 indicates low mood . The cutoff is based on an established cutoff used in a comparable study [4, 5].

The psychometric testing of the GHQ-28 in a general population supported four sub-scales: somatic symptoms, anxiety and insomnia, social dysfunction and severe depression [1]. Psychometric testing of the Norwegian version of the GHQ-28 in a general stroke population confirmed the four-factor solution with an adjusted item loading [6]. In the present study, the analyses and the sub-scales are reported based on the adjusted factor structure of the Norwegian version [6]. The GHQ-28 has been evaluated as an appropriate tool for research purposes for studies that aim to capture psychosocial or emotional distress [2, 3] . The Norwegian version of the GHQ-28 has shown satisfactory measurement properties, reliability and validity [2].

© Bragstad, Hjelle, Zucknick, Sveen, Thommessen, Bronken, Martinsen, Kitzmüller, Mangset, Kvigne, Hilari, Lightbody & Kirkevold.

References

1. Goldberg DP and Hillier VF. A scaled version of the General Health Questionnaire. *Psychol Med* 1979; 9: 139-145.
2. Malt U, Mogstad T and Refnin I. Goldbergs General Health Questionnaire *Tidskr Nor Lægeforen [Journal of Norwegian Medical Association]* 1989; 109: 1391-1394.
3. Goldberg D and Williams P. *A user's guide to the general health questionnaire*. London: Nfer-Nelson, 1991.
4. Watkins CL, Wathan JV, Leathley MJ, et al. The 12-month effects of early motivational interviewing after acute stroke: a randomized controlled trial. *Stroke* 2011; 42: 1956-1961..
5. Watkins CL, Auton MF, Deans CF, et al. Motivational interviewing early after acute stroke: a randomized, controlled trial. *Stroke* 2007; 38: 1004-1009.
6. Hjelle EG, Bragstad LK, Zucknick M, et al. The General Health Questionnaire-28 (GHQ-28) as an outcome measurement in a randomized controlled trial in a Norwegian stroke population. *BMC Psychol* 2019; 7: 18.

© Bragstad, Hjelle, Zucknick, Sveen, Thommessen, Bronken, Martinsen, Kitzmüller, Mangset, Kvigne, Hilari, Lightbody & Kirkevold.
