# Supplementary material for: The effects of a dialogue-based intervention to promote psychosocial well-being after stroke: a randomized controlled trial
Source: Clin Rehabil. 2020 Jun 10;34(8):1056–71. doi: 10.1177/0269215520929737 (PMC7372590; doi:10.1177/0269215520929737)
Supplement: SupplementalFile-2-Imputation – Supplemental material for The effects of a dialogue-based intervention to promote psychosocial well-being after stroke: a randomized controlled trial [file SupplementalFile-2-Imputation.pdf]

Multiple imputation by chained equations (MICE)

In this study, missing values were deemed as missing at random (MAR) [1] and were imputed using multiple imputation by chained equations (MICE). [2] The outcome measures in this study includes several multi-item scales. When single items within the multi-item scales are missing data, it may challenge the types of imputation models that are feasible. [3] In this study, the original imputation model with all the single items from each of the outcome measures was computationally infeasible. Plumpton, Morris, Hughes and White [3] suggests that one way to resolve such issues is to reduce the number of variables in the imputation model by replacing single items with scale summary scores. Thus, we chose to include the sub-scales of GHQ-28 [4] as variables, rather than the 28 single items. The same logic was applied to all the outcome measures with multiple items. The variables that were included in the multiple imputation model are displayed in Table 1.

The imputations were completed in three separate iterations, one for each data collection point of the RCT (T1, T2, T3) (See syntax displayed pp. 3-5). This approach ensures that the missing values are predicted by scores collected at the same time point.

Missing values in the clinical characteristics stroke etiology, stroke localization, and stroke severity (NIHSS) were only imputed in the first iteration (T1) (Table 1) as these values would not change over time. Thus, in the subsequent iterations (T2, T3), the aforementioned variables served as predictors only. For each variable the constraints of the variable were included in the model to ensure imputation within the correct range. The model specified imputation to the closest integer, ensuring that all imputed values were valid values within the range of the original variable. Each imputation model generated 5 imputed data sets based on Rubin's rule. [5]

© Bragstad, Hjelle, Zucknick, Sveen, Thommessen, Bronken, Martinsen, Kitzmüller, Mangset, Kvigne, Hilari, Lightbody & Kirkevold.

The data files from the three separate imputation models were merged before data analysis.

Table 1: Variables included in the multiple imputation model

**Outcomes**	**Variable name**	**Type of scoring**	**Imputation status**	**Constraints - range**
GHQ-28 - Sum	GHQ_CaseScoring_Sum	Case scoring	Predictor + imputation	0-28
GHQ-28 - Somatic sub-scale	GHQ_Likert_Somatic	Likert scoring	Predictor + imputation	0-9
GHQ-28 - Anxiety & insomnia sub-scale	GHQ_Likert_AnxietyInsomnia	Likert scoring	Predictor + imputation	0-33
GHQ-28 – Social dysfunction sub-scale	GHQ_Likert_SocialDysfunction	Likert scoring	Predictor + imputation	0-30
GHQ-28 – Severe depression sub-scale	GHQ_Likert_SevereDepression	Likert scoring	Predictor + imputation	0-12
SAQoL-39g – Physical sub-scale	SAQOLg_Physical	Mean score	Predictor + imputation	1-5
SAQoL-39g – Psychosocial sub-scale	SAQOLg_Psychological	Mean score	Predictor + imputation	1-5
SAQoL-39g – Communication sub-scale	SAQOLg_Communication	Mean score	Predictor + imputation	1-5
Sense of Coherence Scale (SOC-13)	SOC_SumTotal	Sum score	Predictor + imputation	13-65
Lee Fatigue Scale 5	LFS5_Sum	Sum score	Predictor + imputation	5-50
Fatigue Questionnaire, FQ1	FQ1	Binary	Predictor + imputation	0-1
The Yale Brown single item questionnaire	Yale	Binary	Predictor + imputation	0-1
**Baseline demographics & clinical characteristics**	**Variable name**	**Type of scoring**	**Imputation status**	**Constraints - range**
Caring responsibilities	CaringResponsibility	Binary	Predictor	0-1
Rehab services	RehabServices	Binary	Predictor	0-1
Living situation	LivingSituation	Binary	Predictor	0-1
Comorbidities	Comorbidity	Binary	Predictor	0-1
Stroke localization	StrokeLocalization	Categorical	Predictor + imputation (T1)	0-2
Stroke etiology	StrokeEtiology	Binary	Predictor + imputation (T1)	0-1
Sex	Sex	Binary	Predictor	0-1
Age at admission	Age_Admission	Continuous	Predictor	20-90
NIHSS	NIHSS_Total	Continuous	Predictor + imputation (T1)	0-42

© Bragstad, Hjelle, Zucknick, Sveen, Thommessen, Bronken, Martinsen, Kitzmüller, Mangset, Kvigne, Hilari, Lightbody & Kirkevold.

Imputation model (SPSS-Syntax) – Baseline data (T1)

```
DATASET DECLARE Master _Imputation_T1.
MULTIPLE IMPUTATION
GHQ_CaseScoring_Sum
GHQ_Likert_Somatic
GHQ_Likert_AnxietyInsomnia
GHQ_Likert_SocialDysfunction
GHQ_Likert_SevereDepression
SAQOLg_Physical
SAQOLg_Psychological
SAQOLg_Communication
SOC_SumTotal
LFS5_Sum
FQ1
Yale
CaringResponsibility
RehabServices
LivingSituation
Comorbidity
StrokeLocalization
StrokeEtiology
Sex
Age_Admission
NIHSS_Total
/IMPUTE METHOD=AUTO NIMPUTATIONS=5 MAXPCTMISSING=NONE
MAXCASEDRAWS=500 MAXPARAMDRAWS=10
/CONSTRAINTS GHQ_CaseScoring_Sum (MIN=0.0 MAX=28.0 RND=1.0)
/CONSTRAINTS GHQ_Likert_Somatic (MIN=0.0 MAX=9.0 RND=1.0)
/CONSTRAINTS GHQ_Likert_Anxiety (MIN=0.0 MAX=33.0 RND=1.0)
/CONSTRAINTS GHQ_Likert_SocialDysfunction (MIN=0.0 MAX=30.0 RND=1.0)
/CONSTRAINTS GHQ_Likert_SevDepNEW (MIN=0.0 MAX=12.0 RND=1.0)
/CONSTRAINTS SAQOLg_Physical (MIN=1.0 MAX=5.0 RND=1.0)
/CONSTRAINTS SAQOLg_Psychological (MIN=1.0 MAX=5.0 RND=1.0)
/CONSTRAINTS SAQOLg_Communication (MIN=1.0 MAX=5.0 RND=1.0)
/CONSTRAINTS SOC_SumTotal (MIN=13.0 MAX=65.0 RND=1.0)
/CONSTRAINTS LFS5_Sum (MIN=5.0 MAX=50.0 RND=1.0)
/CONSTRAINTS FQ1 (MIN=0.0 MAX=1.0 RND=1.0)
/CONSTRAINTS Yale (MIN=0.0 MAX=1.0 RND=1.0)
/CONSTRAINTS CaringResponsibility (ROLE=IND)
/CONSTRAINTS RehabServices (ROLE=IND)
/CONSTRAINTS LivingSituation (ROLE=IND)
/CONSTRAINTS Comorbidity (ROLE=IND)
/CONSTRAINTS StrokeLocalization (MIN=0.0 MAX=2.0 RND=1.0)
/CONSTRAINTS StrokeEtiology (MIN=0.0 MAX=1.0 RND=1.0)
/CONSTRAINTS Sex (ROLE=IND)
/CONSTRAINTS Age_Admission (ROLE=IND)
/CONSTRAINTS NIHSS_Total (MIN=0 MAX=42 RND=1)
/MISSINGSUMMARIES NONE
/IMPUTATIONSUMMARIES MODELS DESCRIPTIVES
/OUTFILE IMPUTATIONS= Master _Imputation_T1.
```


© Bragstad, Hjelle, Zucknick, Sveen, Thommessen, Bronken,
Martinsen, Kitzmüller, Mangset, Kvigne, Hilari, Lightbody & Kirkevold.

Imputation model (SPSS-Syntax) – 6-month follow-up (T2)

```
DATASET DECLARE Master _Imputation_T2.
MULTIPLE IMPUTATION
GHQ_CaseScoring_Sum
GHQ_Likert_Somatic
GHQ_Likert_AnxietyInsomnia
GHQ_Likert_SocialDysfunction
GHQ_Likert_SevereDepression
SAQOLg_Physical
SAQOLg_Psychological
SAQOLg_Communication
SOC_SumTotal
LFS5_Sum
FQ1
Yale
CaringResponsibility
RehabServices
LivingSituation
Comorbidity
StrokeLocalization
StrokeEtiology
Sex
Age_Admission
NIHSS_Total
/IMPUTE METHOD=AUTO NIMPUTATIONS=5 MAXPCTMISSING=NONE
MAXCASEDRAWS=500 MAXPARAMDRAWS=10
/CONSTRAINTS GHQ_CaseScoring_Sum (MIN=0.0 MAX=28.0 RND=1.0)
/CONSTRAINTS GHQ_Likert_Somatic (MIN=0.0 MAX=9.0 RND=1.0)
/CONSTRAINTS GHQ_Likert_Anxiety (MIN=0.0 MAX=33.0 RND=1.0)
/CONSTRAINTS GHQ_Likert_SocialDysfunction (MIN=0.0 MAX=30.0 RND=1.0)
/CONSTRAINTS GHQ_Likert_SevDepNEW (MIN=0.0 MAX=12.0 RND=1.0)
/CONSTRAINTS SAQOLg_Physical (MIN=1.0 MAX=5.0 RND=1.0)
/CONSTRAINTS SAQOLg_Psychological (MIN=1.0 MAX=5.0 RND=1.0)
/CONSTRAINTS SAQOLg_Communication (MIN=1.0 MAX=5.0 RND=1.0)
/CONSTRAINTS SOC_SumTotal (MIN=13.0 MAX=65.0 RND=1.0)
/CONSTRAINTS LFS5_Sum (MIN=5.0 MAX=50.0 RND=1.0)
/CONSTRAINTS FQ1 (MIN=0.0 MAX=1.0 RND=1.0)
/CONSTRAINTS Yale (MIN=0.0 MAX=1.0 RND=1.0)
/CONSTRAINTS CaringResponsibility (ROLE=IND)
/CONSTRAINTS RehabServices (ROLE=IND)
/CONSTRAINTS LivingSituation (ROLE=IND)
/CONSTRAINTS Comorbidity (ROLE=IND)
/CONSTRAINTS StrokeLocalization (ROLE=IND)
/CONSTRAINTS StrokeEtiology (ROLE=IND)
/CONSTRAINTS Sex (ROLE=IND)
/CONSTRAINTS Age_Admission (ROLE=IND)
/CONSTRAINTS NIHSS_Total (ROLE=IND)
/MISSINGSUMMARIES NONE
/IMPUTATIONSUMMARIES MODELS DESCRIPTIVES
/OUTFILE IMPUTATIONS= Master _Imputation_T2.
```


© Bragstad, Hjelle, Zucknick, Sveen, Thommessen, Bronken,
Martinsen, Kitzmüller, Mangset, Kvigne, Hilari, Lightbody & Kirkevold.

Imputation model (SPSS-Syntax) – 12-month follow-up (T3)

```
DATASET DECLARE Master _Imputation_T3.
MULTIPLE IMPUTATION
GHQ_CaseScoring_Sum
GHQ_Likert_Somatic
GHQ_Likert_AnxietyInsomnia
GHQ_Likert_SocialDysfunction
GHQ_Likert_SevereDepression
SAQOLg_Physical
SAQOLg_Psychological
SAQOLg_Communication
SOC_SumTotal
LFS5_Sum
FQ1
Yale
CaringResponsibility
RehabServices
LivingSituation
Comorbidity
StrokeLocalization
StrokeEtiology
Sex
Age_Admission
NIHSS_Total
/IMPUTE METHOD=AUTO NIMPUTATIONS=5 MAXPCTMISSING=NONE
MAXCASEDRAWS=500 MAXPARAMDRAWS=10
/CONSTRAINTS GHQ_CaseScoring_Sum (MIN=0.0 MAX=28.0 RND=1.0)
/CONSTRAINTS GHQ_Likert_Somatic (MIN=0.0 MAX=9.0 RND=1.0)
/CONSTRAINTS GHQ_Likert_Anxiety (MIN=0.0 MAX=33.0 RND=1.0)
/CONSTRAINTS GHQ_Likert_SocialDysfunction (MIN=0.0 MAX=30.0 RND=1.0)
/CONSTRAINTS GHQ_Likert_SevDepNEW (MIN=0.0 MAX=12.0 RND=1.0)
/CONSTRAINTS SAQOLg_Physical (MIN=1.0 MAX=5.0 RND=1.0)
/CONSTRAINTS SAQOLg_Psychological (MIN=1.0 MAX=5.0 RND=1.0)
/CONSTRAINTS SAQOLg_Communication (MIN=1.0 MAX=5.0 RND=1.0)
/CONSTRAINTS SOC_SumTotal (MIN=13.0 MAX=65.0 RND=1.0)
/CONSTRAINTS LFS5_Sum (MIN=5.0 MAX=50.0 RND=1.0)
/CONSTRAINTS FQ1 (MIN=0.0 MAX=1.0 RND=1.0)
/CONSTRAINTS Yale (MIN=0.0 MAX=1.0 RND=1.0)
/CONSTRAINTS CaringResponsibility (ROLE=IND)
/CONSTRAINTS RehabServices (ROLE=IND)
/CONSTRAINTS LivingSituation (ROLE=IND)
/CONSTRAINTS Comorbidity (ROLE=IND)
/CONSTRAINTS StrokeLocalization (ROLE=IND)
/CONSTRAINTS StrokeEtiology (ROLE=IND)
/CONSTRAINTS Sex (ROLE=IND)
/CONSTRAINTS Age_Admission (ROLE=IND)
/CONSTRAINTS NIHSS_Total (ROLE=IND)
/MISSINGSUMMARIES NONE
/IMPUTATIONSUMMARIES MODELS DESCRIPTIVES
/OUTFILE IMPUTATIONS= Master _Imputation_T3.
```


© Bragstad, Hjelle, Zucknick, Sveen, Thommessen, Bronken,
Martinsen, Kitzmüller, Mangset, Kvigne, Hilari, Lightbody & Kirkevold.

References

1. Jakobsen JC, Gluud C, Wetterslev J, et al. When and how should multiple imputation be used for handling missing data in randomised clinical trials – a practical guide with flowcharts. *BMC Med Res Methodol* 2017; 17: 162.
2. White IR, Royston P and Wood AM. Multiple imputation using chained equations: Issues and guidance for practice. *Stat Med* 2011; 30: 377-399.
3. Plumpton CO, Morris T, Hughes DA, et al. Multiple imputation of multiple multi-item scales when a full imputation model is infeasible. *BMC Res Notes* 2016; 9: 45.
4. Hjelle EG, Bragstad LK, Zucknick M, et al. The General Health Questionnaire-28 (GHQ-28) as an outcome measurement in a randomized controlled trial in a Norwegian stroke population. *BMC Psychol* 2019; 7: 18.
5. Rubin D. *Multiple Imputation for Nonresponse in Surveys*. New York: John Wiley and Sons, 2004.

© Bragstad, Hjelle, Zucknick, Sveen, Thommessen, Bronken,
Martinsen, Kitzmüller, Mangset, Kvigne, Hilari, Lightbody & Kirkevold.
