# Supplementary material for: The effects of a dialogue-based intervention to promote psychosocial well-being after stroke: a randomized controlled trial
Source: Clin Rehabil. 2020 Jun 10;34(8):1056–71. doi: 10.1177/0269215520929737 (PMC7372590; doi:10.1177/0269215520929737)
Supplement: SupplementalFile-3-LMM – Supplemental material for The effects of a dialogue-based intervention to promote psychosocial well-being after stroke: a randomized controlled trial [file SupplementalFile-3-LMM.pdf]

Linear Mixed Model analysis

A linear mixed model (LMM) was used to assess the primary outcome emotional distress at 12 months post-stroke, measured by the General Health Questionnaire-28 (GHQ-28). The dependent variable was the GHQ-28 sum score, using the Likert scoring method (Supplementary file 1). Sensitivity analyses [1] were conducted to explore whether "recruiting hospital", the "intervention providers' professional background", or "data collector ID" should be included as random effects. The sensitivity analyses concluded that none of the additional variables should be included in the model. The model fit was better without including the additional random effect variables. The final LMM included 14 fixed effects variables, and 1 random effect variable (Table 1).

Table 1: Overview of variables included in the final LMM

**Dependent variable**	**Fixed effects variables**	**Random Effects Variable**
GHQ_Likert_sum	Time	Time
	GroupAllocation	
	Sex	
	Age_Admission	
	StrokeEtiology	
	StrokeLocalization	
	NIHSS_Total	
	LivingSituation	
	Comorbidity	
	RehabServices	
	CaringResponsibilities	
	Yale	
	FQ1	
	SOC_SumTotal	

As the LMM analysis was performed on a multiply imputed data file with 5 imputations, the analysis (See syntax p 2.) was performed separately for each of the imputations (1-5). The results were subsequently pooled across all 5 imputations using the statistical software R v3.6.1 [2] with package mitools v2.4.

© Bragstad, Hjelle, Zucknick, Sveen, Thommessen, Bronken, Martinsen, Kitzmüller, Mangset, Kvigne, Hilari, Lightbody & Kirkevold.

SPSS Syntax for LMM

```
GENLINMIXED
/DATA_STRUCTURE SUBJECTS=PatientID REPEATED_MEASURES=Time
COVARIANCE_TYPE=DIAGONAL
/FIELDS TARGET=GHQ_Likert_sum TRIALS=NONE OFFSET=NONE
/TARGET_OPTIONS DISTRIBUTION=NORMAL LINK=IDENTITY
/FIXED  EFFECTS=Time GroupAllocation Sex Age_Admission StrokeEtiology StrokeLocalization
NIHSS_Total LivingSituation Comorbidity RehabServices CaringResponsibilities Yale FQ1
SOC_SumTotal USE_INTERCEPT=TRUE
/RANDOM EFFECTS=Time USE_INTERCEPT=FALSE SUBJECTS=PatientID
COVARIANCE_TYPE=AR1 SOLUTION=FALSE
/BUILD_OPTIONS TARGET_CATEGORY_ORDER=DESCENDING
INPUTS_CATEGORY_ORDER=DESCENDING MAX_ITERATIONS=300
CONFIDENCE_LEVEL=95 DF_METHOD=RESIDUAL COVB=ROBUST
PCONVERGE=0.000001(ABSOLUTE)
   SCORING=0 SINGULAR=0.000000000001
/EMMEANS TABLES=Time COMPARE=Time CONTRAST=PAIRWISE
/EMMEANS_OPTIONS SCALE=ORIGINAL PADJUST=LSD.
```

References

1. Jakobsen JC, Gluud C, Wetterslev J, et al. When and how should multiple imputation be used for handling missing data in randomised clinical trials – a practical guide with flowcharts. *BMC Med Res Methodol* 2017; 17: 162.
2. R Core Team. R: A language and environment for statistical computing. Vienna, Austria. R Foundation for Statistical Computing, 2019.

© Bragstad, Hjelle, Zucknick, Sveen, Thommessen, Bronken, Martinsen, Kitzmüller, Mangset, Kvigne, Hilari, Lightbody & Kirkevold.
