# Supplementary material for: The effects of a dialogue-based intervention to promote psychosocial well-being after stroke: a randomized controlled trial
Source: Clin Rehabil. 2020 Jun 10;34(8):1056–71. doi: 10.1177/0269215520929737 (PMC7372590; doi:10.1177/0269215520929737)
Supplement: Suppl_Table_S1 – Supplemental material for The effects of a dialogue-based intervention to promote psychosocial well-being after stroke: a randomized controlled trial [file Suppl_Table_S1.pdf]

Supplementary Material

Table S1: Outcomes and measures with scoring and time of assessment.

**Primary outcome**	**Measure**	**Description**	**Scoring**	**Assessment**[1]
Psychosocial well-being	The General Health Questionnaire-28 (GHQ-28) [17, 18]	Scaled 28-item self-report questionnaire measuring emotional distress. Four sub-scales identified in psychometric tests (somatic symptoms, anxiety and insomnia, social dysfunction and severe depression).[17, 26] Likert scoring, items ranging from 1-4.<br><br>Case scoring, items ranging from 0-1.	Range sum Likert scoring: 0-84, lower score indicates lower level of distress.<br><br>Range sum Case scoring: 0-28, cutoff at 5; < 5 indicates normal mood, and ≥ 5 indicates low mood.	T1, T2, T3
**Secondary outcomes**	**Measure**	**Description**		**Assessment**
Health-related quality of life	Stroke and Aphasia Quality of Life Scale-39 generic stroke version (SAQOL-39g) [41, 42]	Self-report 39-item stroke-specific health-related quality of life scale. Measures patient's perspective of stroke's impact on 'physical', 'psychosocial' and 'communication' domains. Likert scoring, items ranging from 1-5.	Range mean score: 1-5<br><br>Higher mean score indicates higher functioning; higher quality of life score.	T1, T2, T3
Sense of coherence	Sense of Coherence scale (SOC-13) [28]	Self-report questionnaire, 13 items measuring the main concepts in the sense of coherence theory;	Sum range: 13-65.<br><br>Higher scores indicate a stronger sense of coherence.	T1, T2, T3

[1] T0=Data from acute phase collected from patient record, T1=Baseline assessment at 4-6 weeks post-stroke, T2=Assessment at 6 months post-stroke, T3=Assessment at 12 months post-stroke

		coherence, meaningfulness and manageability. Likert scoring, ranging from 1–5.		
Depression	The Yale Brown single item questionnaire (Yale) [43, 44]	Self-reported presence or absence of depression.	Yes/No	T1, T2, T3
**Characteristics of sample**	**Measure**	**Description**		**Assessment**
Fatigue	Fatigue Questionnaire-2 (FQ-2) [45, 46]	Self-reported presence or absence of fatigue.<br><br>If yes; indication of duration of symptoms.	Yes/No	T1, T2, T3
Aphasia	The Ullevaal Aphasia Screening Test (UAS) [47]	Screening for aphasia. Based on scores and clinical judgement, 4 categories: 1) No language impairment, 2) Mild language impairment, 3) Moderate language impairment, 4) Severe language impairment.	Range 0-52, scores <50 indicate pathologic language functioning.[48]	T1
Stroke severity/ neurological deficit	National Institutes of Health Stroke Scale (NIHSS) [49]	An 11-item scale used by healthcare providers to objectively quantify the impairment caused by a stroke.	Range 0-42.<br><br>Cutoffs: 0-5=Mild symptoms of stroke, 6-10= moderate symptoms of stroke, ≥11=Moderate to severe stroke symptoms.	T0
Cognitive function	Mini Mental State Evaluation (MMSE) [50]	30-point test that is used to measure potential cognitive impairment.	Range 0-30.<br><br>Cutoff at 24 to indicate cognitive impairment. A score below 24 indicates cognitive impairment ranging from mild (19-23), moderate (10-18), and severe (≤ 9).	T0
